# Round the Hospitals

**Published:** 1919-11-01

**Authors:** 


### ROUND THE HOSPITALS.

Every member of the College whose honour has been assailed by the wild women of Mortimer Hall should bestir themselves and uphold the College of Nursing, by each of them resolving to render continuous personal service in its defence, for the next three months. There are at present in round numbers 16,000 nurses, qualified to be registered the moment the Bill is passed, who are members of the College of Nursing and who might each resolve to secure between now and the middle of January, at least one fully trained nurse as a new member of the College, and so bring up the total

**ROUND THE HOSPITALS—(*continued*).**

number of College members qualified for immediate registration to 32,000. Every intelligent and sound nurse who has an especial value for each patient entrusted to her care will be benefited by raising the members on the College Register without further delay to a minimum of at least 20,000. It should be neither difficult, nor a time-absorbing task, for any of the present members of the College, to find and safely conduct one of her sisters to Miss Rundle and to see her name duly entered on the College Register. All things come round to her who can but wait, and the days of waiting for College nurses are now so reduced in volume, that if all will act together, they can be eaten up by the new members who will become attached to the College before the last day of 1919 arrives in due course. Wake up, College members. Show your mettle. Down with the wild women, through whose machinations and those of a few self-seeking irresponsibles harm might come, and take steps to save nurses who have lost their equilibrium through the excitement of the war and demobilisation, who have torn up the teachings of Florence Nightingale, and at the moment may feel a desire to join a Trade Union. Such a step must disqualify them permanently for the career and responsibilities of a fully trained nurse. We are confident that no fully trained nurse worthy of the name will ever commit her professional suicide by handing over her freedom of action and personal liberty to any clique which may promote yet another association and denominate it A Trade Union for Nurses. Let every nurse realise the folly of such a step and be wise.

As we go to press there is still no sign that the authorities of Queen Charlotte's Hospital are willing, and it is therefore possibly just to assume that they are unable, to make any radical changes or to take steps to show their willingness and desire to convert that institution into an up-to-date, well-equipped, excellent training-school for midwives who are going to practise. We have it on authority that Queen Charlotte's very rarely trains practising midwives. This fact may account for their apparent indifference to the great need for an excellent, well-equipped, up-to-date, big training-school for midwives who are going to practise and get their living in this field. Such a school, to fulfil the needs of the profession and the public, must be encouraged in some way to train midwives, not monthly nurses, and to ensure that the pupils so trained shall practise as midwives.

The discussion which has so far arisen in regard to the state of affairs at Queen Charlotte's Hospital seems to have inspired activity in connection with the proposed National Training-school for district midwives at Woolwich, the building for which is to be begun in March 1920. The new post-graduate training-school too, connected with the York Road Hospital, has set an excellent example, and is undoubtedly a valuable development. A graduate training-school on the lines of that connected with the York Road Hospital has long been a need, and we wish it every possible success.

We have had very strong confirmation of the great need too for an excellent, up-to-date, well-equipped and large training-school for midwives who intend to practise as a means of obtaining an adequate livelihood. The openings for trained midwives of the best are continually increasing under existing conditions, and should prove sources of good income to the women who decide to follow this branch of work. It gives us pleasure to add the testimony of those who have had to do with him, that Mr. Watts, the secretary of Queen Charlotte's Hospital, is always most kind and pleasant to those who have any business to transact with him. Cannot he use these qualities to secure that the necessary steps shall be taken with all possible speed to raise Queen Charlotte's Hospital out of the rut and discredit into which it appears to have fallen?

At the Investiture held by the command of the King in the Throne Room, St. James's Palace, on October 22, Lieut.-Colonel Prince Arthur of Connaught conferred the following decorations on members of the nursing services on his Majesty's behalf: Royal Red Cross, First Class, Sister Lilian Terrill, Q.A.I.M.N.S. (R.); Royal Red Cross, Second Class, Miss Gertrude Blacklock and Miss Annie Palmer, Q.A.I.M.N.S. (R.); Mrs. Helen Pyper, B.R.C.S.

By permission of the King, St. James's Palace was the scene of a very important Conference, held on October 23, to discuss the future constitution of Voluntary-Aid Detachments. The Ministries of Health and Pensions have drawn up a scheme through which voluntary and part-time workers may be utilised in peace. The Detachments are to remain a part of the Technical Reserve of the Territorial Force. Their management is delegated by the War Office to a Joint Council which in its turn will set up a Central Joint V.A.D. Committee. An influential company assembled to assist at the Conference, including Sir Arthur Stanley, Chairman, Dr. Addison, Minister of Health, and Colonel Webb, Director-General Medical Service, and numbered also Princess Beatrice, the Duchess of Beaufort, the Duchess of Norfolk, and many other notables who testified by their presence the interest they took in the work thus inaugurated. Dame Sarah Swift represented the nursing side of the Conference.

Among the decorations and medals awarded by the Allied Powers at various dates to the British Forces for distinguished services rendered during the course of the campaign are the following awarded to members of the nursing services:—Conferred by the King of the Belgians: *Medaille de la Reine Elizabeth*—Acting Sister E. E. Appleton, Sister A. I. Baird, Mrs Barron, Acting Sister A. M. Browett, Acting Sister E. E. Cooke, Sister E. M. Cooper,

**ROUND THE HOSPITALS**—*(continued).*

Acting Sister C. E. Druce, Sister C. Elston, Sister F. H. Freshney, Acting Sister K. E. Haywood, Sister A. M. Heffernan, Sister B. M. Huddlestone, Acting Matron I. Johnson, Sister E. F. Jones, Sister M. T. Lynch, Acting Matron M. McCormick, Acting Sister A. A. Mate, Acting Sister C. F. Middleton, Acting Sister F. A. Morgan, Acting Sister E. Pilkington, Acting Sister A. Reburn, Acting Matron M. H. Smythe, Acting Sister F. A. Spedding, Sister M. R. Stewart-Richardson, Miss A. M. White, Acting Matron I. M. Whyte, Acting Matron A. P. Wilson. *The Order of Leopold II, Chevalier,* has been conferred on four members of the First Aid Nursing Yeomanry Corps: Miss B. Ellis, Miss G. Marples, Miss M. Moseley-Williams, Miss V. C. N. Power. Conferred by the King of Serbia: *Order of St. Sava,* 5th Class: Matron A. B. Cameron, Matron J. M. Clay, Acting Matron M. Jones, Matron A. M. Milligan, R.R.C., Matron J. Smales. *Gold Medal for Valour*: Sister A. R. Colhoun, Sister E. Garrett. *Samaritan Cross*: Staff Nurse E. E. Allen, Staff Nurse B. E. Barrett, Sister E. M. Broome, Sister A. Brown, Sister C. L. Cracknell, Staff Nurse R. C. Crisford, Sister H. Douglas, Sister A. E. Grant, Sister G. E. Green, Assistant Matron E. Harwood, Sister J. Holforth, A.R.R.C., Sister E. Lee, Staff Nurse G. L. Line, Staff Nurse A. Macleod, Staff Nurse F. S. Major, Sister J. T. Mitchell, Sister E. Percy, Sister S. G. Rees, Sister H. M. Ripper, Sister C. M. Robinson, Sister H. D. Simpson, Sister H. Smith, Staff Nurse A. Ward, Sister A. K. Wheller, Sister B. Wotherspoon. The King has given unrestricted permission in all cases to wear the decoration and medals in question.

Additional instructions have now been issued on the issue of Army of Occupation bonus to nurses. This bonus is granted under Army Order 300 of 1919 to certain members of the Q.A.I.M.N.S.R., T.F.N.S., and to certain V.A.D. nursing members, special military probationers, and assistant nurses, and temporary nursing staffs of military families' hospitals necessarily retained on military service. Until further notice a bonus at the same rates as those approved for reserve and temporary staffs is granted to members of the Q.A.I.M.N.S. and of the permanent nursing establishment of military families' hospitals. The bonus is based on the rank, acting or substantive, for which pay is being drawn. Acting rank specially granted without pay and allowances of the rank will not entitle the holder to the higher rate for such rank. In cases other than those of the Q.A.I.M.N.S. and the permanent nursing establishment of the military families' hospitals, the bonus is only issued subject to the nurse having signed a contract to continue to serve until April 30, 1920, or until her services are no longer required, whichever is the earlier date. To draw the bonus from February 1, 1919, nurses and V.A.D. nursing members serving at home must sign A.F.W. 5120 before November 1 next, and those serving overseas must complete A.F.W. 5120 within one month from the date on which A.C.I. 591 is received in the area in which they are serving. Nurses and V.A.D. nursing members who sign the agreement after the dates above specified are eligible for the bonus only from the date of signing A.F.W. 5120. Nurses and V.A.D. nursing members on sick leave should complete the form within one week of the date of rejoining for duty. They will then be eligible for the bonus from February 1.

A delightful holiday has lately been enjoyed by the one hundred members of Q.A.I.M.N.S., the Reserve, and the T.F.N.S., who accepted the invitation of Surgeon-General Daae and his coadjutors to spend a month in Norway. The sisters went in parties of ten, and were entertained most generously and hospitably by members of the Norwegian Committee, who did all in their power to make the sojourn restful and happy. One of them relates a visit to the King and Queen of Norway at their country house, where they were received with royal kindness. They were particularly charmed with the welcome accorded them by Queen Maud, who gave them tea, and at meeting Prince Olaf. Surgeon-General Daae must be aware that this spontaneous tribute from Norway to British nurses has brought the two countries appreciably together, and set a current of sympathy stirring which will have widespread vibrations.

The luncheon party given by one hundred representative women to the heads of the uniformed Auxiliary Services was a brilliant function from first to last. Princess Alice, Countess of Athlone, was the hostess of honour and the Queen showed her sympathy by sending flowers for the tables. The guests were Lady Ampthill, V.A.D.s; Lady Londonderry, Women's Legion; Dame Florence Barleigh Leach, Q.M.A.A.C.; Mrs. Gwynne Vaughan, W.R.A.F.; Miss Muriel Talbot, Land Army; and Dr. Flora Murray, Endell Street Hospital; Dame Katherine Furse, W.R.N.S., was unable to attend. Dame Sidney Browne was also present. Miss Lena Ashwell read a charming address of welcome to the guests, composed by Mrs. Meynell.

At the meeting of the London Insurance Committee on October 23, the General Purposes Sub-Committee reported that they have reconsidered the provision of nursing services for insured persons. They understand that there are thirty-one associations under the auspices of the Central Council for district nursing in London, and that such associations employ 225 fully-qualified nurses. It is computed that the nurses pay 3,000 visits a year each, and from the figures available it appears that in the case of the Ranyard nurses the number of visits paid to insured persons represents from 18 to 20 per cent. of the total visits paid by the nurses; while in the case of the Queen's nurses the proportion is about 12 per cent.

**ROUND THE HOSPITALS**—(*continued*).

The Central Council was formed to provide complete nursing services all over London. The Committee understand that the Council has not fully achieved its object, and that there are certain gaps which it is inadequate to fill. The Council is urgently in need of funds, and it has considered very carefully whether it is possible for the Committee to make a grant in this connection. The Committee are of opinion that the Insurance Committee might make a grant of £500 to the Central Council, and there was a recommendation to this effect, but the final decision was deferred.

---

The meeting held on October 25 to form a trade union for trained women nurses carried the proposal by a large majority. The objects of the union are familiar enough. They are promotion of State Registration, establishment of an employment agency, the securing of a minimum rate of remuneration and maximum working hours, the provision of benefits for members when totally incapacitated, and the abolition of abuses detrimental to their welfare and economic independence. What is not yet apparent is the methods through which the promoters of the undertaking propose to carry through this programme. These are reforms for which thousands of nurses are already zealously working, and it might have occurred, we think, to Miss Maude M. Callum and Miss Macdonald, who took the lead after their solicitor in urging the company to combine, that union is strength. We await with some interest the disclosure of the rules which will bind members of the new association.

---

The National Food Reform Association has amalgamated with the Food Education Society, and for the future will be known under the latter name. It issues accounts for the years 1914 to 1918 inclusive, with a rather vague and diffuse report for the same period, recounting its activities during wartime. Its publications appear to have been a good deal in request. Lectures and demonstrations on cookery have been given from time to time, and propaganda in favour of food reforms has been carried through from the headquarters of the Society. It has now been registered under the Board of Trade as an "Association not for profit," and has moved its office to Dane's Inn House, 265 Strand, W.C. 2.

---

It would be a calamity if the movement for at last founding a worthy Nurses' Home at St. Bartholomew's should be in any way damped down by the appeal for the larger necessities of the hospital. Could not subscribers be asked to ear-mark part of their contributions for the nurses? We notice that the benefits conferred on the public by the training at St. Bartholomew's of skilled nurses is prominently alluded to in the appeal. It would quicken the response could visitors to this world-renowned institution be taken round the squalid quarters in Little Britain, where at present, and for many long years past, the nursing staff has endured manifold discomforts.

---

It appears that the Administrator of Cape Town has, at least temporarily, refused to consider the proposal to raise the salaries of Cape nurses to the level reached in the Transvaal. Staff nurses at the Cape receive £65 and sisters £120, plus board, lodging, and uniform. This, it is contended by the *South African Nursing Record*, is not a living wage, and the rather amusing comment follows: "We hear so much of nurses marrying after qualification. Is it to be wondered at? Any outlet would seem to be justified." But after all are nurses driven to matrimony simply and solely because on an income of £5 8s. 4d. per month, "it takes two months' pay to buy a decent evening dress"?

---

Queen Mary's Maternity Home at Hampstead, now approaching completion, takes its rise, as many of our readers will remember, from the Queen's Needlework Guild, the balance of the funds, £30,000, having been devoted to this object. Cedar Lawn, on Hampstead Heath, is the temporary building where the Home will open next month. But a fine site near the Heath, called The Paddock, has been generously presented to the Queen for the erection of the permanent Home when building is possible. There is to be accommodation for about twenty cases, and special arrangements for middle-class mothers who may desire to pay their own expenses. Quarters are being arranged for little children whom the mothers could not leave behind at home, an experiment which is succeeding well at Plymouth. The Hampstead Home, planned on the simplest lines, yet with a comprehensive grasp of the needs of both mothers and infants, is likely to become a model for local authorities and command widespread imitation. Members of the Needlework Guild will see to it that nothing is lacking which their fingers can produce, and it starts under the happiest auspices with the announcement that no annual subscription will be required though donations will be very acceptable.

---

The blind masseurs, trained for the most part under the ægis of St Dunstan's and the National Institute for the Blind, have formed an association of their own and celebrated its inauguration in the comfortable British fashion by dining together. Over fifty, some of whom were civilians, assembled at the Holborn Restaurant under the presidency of Sir Arthur Pearson, and Sir Bruce Bruce-Porter said of them to their face that all those present were heroes in the best sense of the word, as their achievement and their courage proved beyond question. The rapid extension of massage among those who have lost their sight testifies to the existence of a sixth sense which makes the blind peculiarly sensitive to what comes under their touch and peculiarly dexterous in manipulation. We heartily wish the new association Godspeed.

